# Empirical lessons regarding contraception in a protracted refugee setting: A descriptive study from Maela camp on the Thai-Myanmar border 1996 – 2015

**DOI:** 10.1371/journal.pone.0172007

**Published:** 2017-02-23

**Authors:** Somjet Srikanok, Daniel M. Parker, Amber L. Parker, Tracey Lee, Aung Myat Min, Pranee Ontuwong, Saw Oo Tan, Supachai Sirinonthachai, Rose McGready

**Affiliations:** 1 The Planned Parenthood Association of Thailand, Bangkok, Thailand; 2 Shoklo Malaria Research Unit, Mahidol-Oxford Tropical Medicine Research Unit, Faculty of Tropical Medicine, Mahidol University, Mae Sot, Thailand; 3 Centre for Tropical Medicine and Global Health, Nuffield Department of Medicine, University of Oxford, Old Road campus, Oxford, United Kingdom; University of Ottawa, CANADA

## Abstract

Conflict settings and refugee camps can be chaotic places, with large and rapid population movements, exacerbated public health problems, and *ad hoc* health services. Reproductive health care that includes family planning is of heightened importance in such settings, however, funding and resources tend to be constrained and geared towards acute health services such as trauma management and infectious disease containment. Here we report on the complexities and challenges of providing family planning in a post-emergency refugee setting, using the example of the largest refugee camp on the Thai-Myanmar border, in existence now for over 30 years. Data from 2009 demonstrates an upward trend in uptake of all contraceptives, especially long acting reversible contraception (LARC) and permanent methods (e.g. sterilization) over time. Increased uptake occurred during periods of time when there were boosts in funding or when barriers to access were alleviated. For example a surgeon fluent in local languages is correlated with increased uptake of tubal ligation in females. These data indicate that funding directed toward contraceptives in this refugee setting led to increases in contraceptives use. However, contraceptive uptake estimates depend on the baseline population which is difficult to measure in this setting. As far as we are aware, this is the longest reported review of family planning services for a refugee camp setting to date. The lessons learned from this setting may be valuable given the current global refugee crisis.

## Introduction

Worldwide, an estimated 289,000 women died during pregnancy or childbirth in 2013. The overwhelming majority of these deaths were in disadvantaged populations, in rural and remote areas, and in the worlds’ poorest nations [1]. Most could have been prevented. An estimated 225 million women want but are unable to acquire contraceptives and approximately one out of every three deaths related to pregnancy and childbirth might have been prevented if there had been adequate access to contraceptives [2].

Conflict areas disproportionately contribute to global maternal mortality [3–5]. This has led to increasing advocacy for reproductive health to be a seriously recognized element of humanitarian responses [6], with variable success [7]. Barriers to success are related to insufficient aid for reproductive activities [8], lack of institutional capacity [7], socio-cultural barriers [9], lack of commodity management and security resulting in inconsistent stocks of essential supplies, lack of appropriate engagement with local communities [9] and lack of accurate data (including baseline population numbers [10]). Reproductive health services (incorporating medical care, education and contraceptives) are crucial in these settings, not only with regard to maternal mortality but also with regard to child mortality, [11] morbidity [12] and poverty [13]. In refugee settings mortality is highest among children [11]. Poor health in pregnant women can lead to poor fetal and child health [14] and some research suggests that the fetal environment can have profound influences on health throughout the lifespan [12]. There is also a growing body of evidence regarding intergenerational poverty cycles whereby mothers who are chronically ill or who live in poverty have an increased likelihood of raising children who also go on to be chronically ill or live in poverty in their adulthood and reproductive careers [13,15]. Contraception directly benefits mothers and children and also boosts the health and wellbeing of entire communities, including refugees and refugee settlements [16,17].

Recent evidence suggests that long acting reversible contraceptives (LARC), and permanent methods such as sterilization, can be offered in conflict settings despite logistical concerns, and are especially advantageous in crisis situations [16]. They provide long-lasting protection against unwanted pregnancy without dependency on regular availability, which can be interrupted by sudden or unexpected increases in demand, for example, through an influx of refugees due to disaster or conflict, funding issues, or physical disruptions in supply because of damaged transport networks. LARC and permanent methods offer effective alternatives for couples where pill and condom efficacy are heavily influenced by compliance and appropriate storage or immediate availability at each sexual encounter. They lessen logistical burdens for women and men with regard to travel time and distance to acquire contraceptives on a regular basis. Furthermore, both LARC and permanent methods are discreet and may help to alleviate stigma or perceived stigma around purchasing contraceptives on a regular basis, perhaps especially among adolescents and those engaged in sexual activity outside marriage.

Here we report on population estimates, contraceptive services, and birth outcomes in Maela refugee camp on the Thai-Myanmar border over a 19-year time period. This work aims to highlight the potential benefits and rewards of providing contraceptives as well as the difficulty of collecting basic demographic data in resource-limited settings. As far as we are aware, this is the longest reported review of family planning services for a refugee camp setting to date.

## Methods

### Background to this setting

#### Maela camp

In the early 1980s several small refugee and internally displaced person (IDP) camps emerged along the approximately 2000km long international border between Thailand and Myanmar as people fled conflict in Myanmar (then officially named Burma) [18]. Many of these small village-sized camps were eventually consolidated into much larger camps in the late 1990s [18]. Maela camp was one of these consolidation camps and the population changed rapidly from an estimated 6,000 inhabitants in 1994 to 31,000 in 1998, growing to almost 50,000 in 2006, and is now approximately 38,000 as of December 2015, (based on Thai-Burma Border Consortium estimates) [19]. The camp is approximately 54 km from the next major town (Mae Sot, Tak Province, Thailand) and 20 km from the nearest Thai hospitals (Tha Song Yang Hospital to the north and Mae Ramat hospital to the south). The camp is ethnically diverse, but is primarily composed of the Karen minority group (mostly S’gaw and Pwo) as well as other minority ethnic groups from Myanmar. The main languages spoken in the camp are S’gaw Karen and Burmese followed by Pwo Karen.

#### Health and family planning services

Originally the main health provider in Maela camp was Médecins Sans Frontières (MSF) who withdrew in May 2005 handing over their camp facilities to Aide Medicale International (AMI). In 2011 AMI merged with Première Urgence (PU) creating PU-AMI who remained the major health provider in the camp until 2015. Commencing in 1986 Shoklo Malaria Research Unit (SMRU) initially conducted antenatal clinics (ANC) and birthing services alongside MSF with the original goal of preventing maternal death from malaria [20]. After discussions with MSF, SMRU became the sole provider of ANC and delivery services, including post-abortion care, in Maela camp in 1997.

Prior to 1991 women had access to a limited range of contraceptives through MSF medical outpatient clinics, including monophasic combined oral contraceptive pills (0.03 mg ethinyloestradiol (COCP)) and depomedroxyprogesterone acetate (150 mg, injected on a 12 weekly basis (Depo-injections)) but no family planning promotion or education and only brief counseling was offered in the outpatient department.

SMRU’s family planning program began in May of 1996 in one of the original camps along the border (Shoklo) and in Maela refugee camp after consultation and permission from local Karen camp administrators. The amalgamation of the camps in the late 1990s into Maela required sudden expansion and increased demand on services and staff. For convenience, the SMRU family planning clinic in Maela was located adjacent to the ANC and birthing room and was staffed by trained male and female health workers. It provided a range of contraceptives as well as education and counseling to women and men, regardless of age and marital status. Contraceptive options included: natural methods, condoms, COCP, emergency contraception pills (ECPs), Depo-injections, Implant, Intra Uterine Device (Multiload—250^®^); vasectomy in the camp and tubal ligation via mini-laparotomy in the Thai Public Hospital. The progestin only pill was not available in Thailand at the start of the program but it was made available from Family Planning Australia^®^ (FPA) but uptake was poor and it was not continued. In 1996 Norplant^®^ was the only implant available and in 2004 it was replaced by Implanon^®^. In 1996 we were unable to find any report of Norplant^®^ use in refugees and a concerted effort was made to follow-up these women [21]. Misoprostol is not easily available in Thailand to humanitarian aid organizations. In early pregnancy SMRU uses misoprostol for treatment of fetal demise, annovulatory pregnancy and incomplete abortion; all confirmed by ultrasound. It is not used as an abortificant due to restrictive abortion laws in Thailand [22]. Rates of sexually transmitted infections (STIs) in Maela refugee camp, first examined by laboratory screening in 1997, are very low and there is no evidence they have increased over time in this population [23,24].

In April 2000, the Planned Parenthood Association of Thailand (PPAT) was funded and committed to provide comprehensive family planning services within Maela. In order to prevent service duplication, the SMRU core family planning staff and all SMRU clients and records were handed over to PPAT. PPAT reproductive health activities in the camp are significant and include family planning counseling to all post-partum women; outreach reproductive health education for men, women and adolescents in the camp and gynecological care (examination of breast and vaginal discharge, PAP smear, infertility and irregular menstruation counseling services). Reproductive health education from PPAT targeted men in order to promote male involvement in family planning, included women’s training that focused on sexual and reproductive health and rights as well as peer education for adolescents. PPAT staff and volunteers also received specific training on ECPs.

SMRU continued to conduct vasectomy procedures in the camp until PPAT took over the provision of this service in April 2001. From 2005 through 2007 SMRU employed a surgeon who, with the assistance of a local midwife, established a female tubal ligation service on site, overcoming the need for referral to the Thai Public Hospital system [21]. Post-partum tubal ligation was the preferred method although interval sterilization was provided as needed. After the surgeon left SMRU (in 2007) provision of camp based sterilization service was only available when the attending obstetric doctor had that skill. PPAT were also able to refer to the Thai Public Hospital system.

Collaboration between the organizations has continued with PPAT intermittently conducting education at SMRU ANC and SMRU sharing birth lists of camp residents with PPAT who would conduct individualized follow-up visits. Also, PPAT collated monthly and yearly data for the whole camp while SMRU provided data on tubal ligation to PPAT and later on IUD.

#### Funding for contraceptives and family planning

Supply of contraceptives was initially fully supported by the Thai Department of Public Health (TDPH). The TDPH welcomed Karen staff from the refugee camp to receive training for Norplant insertion at a local hospital (Tha Song Yan Hospital). However, as a result of the 1997 Asian financial crisis [25] the TDPH was unable to continue to supply contraceptives. SMRU then sought funding elsewhere and FPA were able to provide an emergency budget for contraceptive supply for 36 months. PPAT receives funding in part from the Patronage of Her Royal Highness the Princess Mother of Thailand (www.ppat.or.th/en/about_strategy). Fluctuations in global and local economics as well as political sentiment have adversely affected International PPA (IPPA) and hence PPAT operations. In 2011 a donation from Malariadoktor Foundation and Stichting Vluchteling supported PPAT, and SMRU received a donation from the Dayalu Foundation in 2015. PPAT has at times asked for donations from clients who can afford to pay to help cover the costs of contraceptives.

### Data

To quantify the uptake of contraceptives and new family counseling visits, rates (e.g. use per 1,000 reproductive age women, per year) were calculated with the denominator being the baseline population. Pinpointing the current or yearly population can be difficult in refugee camps and resulting rates and figures require cautious interpretation [10]. Different agencies within refugee camps can provide overlapping services, so that isolating exact numbers without duplicate counting can be problematic. Maela camp is a closed population, meaning that residents are unable to legally travel within Thailand, however, the reality is much more complex [26] with refugees travelling out of the camp for economic purposes, in both legal and illegal capacities. Furthermore many villagers (non-camp residents) travel to the camp for health care and sustenance, as it is a source of food and basic medical services.

Two main organizations have kept population estimates for the camp: the Thai-Burma Border Consortium (TBBC) since 1998 and the United Nations High Commissioner for Refugees (UNHCR) since 2002. The TBBC provides services to refugees including some support for housing and food rations based on population counts [27]. TBBC numbers include camp dwellers who are not officially registered by UNHCR. UNHCR numbers started well after Maela camp was established and have systematically been lower than TBBC numbers but may be influenced by political sentiments; in this case they may have been influenced by real or perceived aggravation by Thai authorities regarding a growing, foreign, uninvited population, as with refugees elsewhere [28].

Since 1998, TBBC has reported monthly population estimates that include UNHCR estimates. We used population estimates from December of each year from both TBBC (1998 through 2015) and UNHCR (2002 and 2015).

### Contraceptive uptake data

PPAT maintains monthly records of family planning consultations, including new and ongoing contraceptive consultations as well as house visits by PPAT for family planning counseling. “New” consultations are newly registered women to the service (SMRU or PPAT) and include women who started a method of contraception for the first time but not those who switched from a different method or had a gap in between using contraceptives.

### Births and miscarriages per year

The vast majority of births in Maela camp occur at SMRU. All pregnant women providing a Maela address at ANC enrollment were included in the analysis. Pregnancy outcomes were noted regardless of place of pregnancy outcome and include live- and still-birth, miscarriage, or those lost to follow-up before pregnancy outcome was available (usually because of migration).

### Analysis

For most years the population data are split by sex but not stratified by age. For years in which population by sex was not reported we estimated 49.79% of the population as female, using the average percentage of females for years in which the population by sex is reported. We then estimated the proportion of the female population who were of reproductive age (between the ages of 15 and 44) as 41.66% based on demographic surveillance systems in near-by Karen villages on the Thai side of the border [29].

We created smoothed population estimates by fitting a loess curve (a nonparametric locally weighted polynomial regression approach [30]) to the data points and extracting the smoothed values using R cran 3.2. These smoothed estimates were used as denominators for calculating the cumulative uptake of LARC and tubal ligation as well as the pregnancy and miscarriage rates. We used the smoothed population estimates from both TBBC and the UNHCR as a sensitivity analysis for our estimates of LARC and tubal ligation uptake, pregnancy rate and the miscarriage rate.

We calculated all new: contraceptive consultations; total pregnancies; and miscarriages for a given year per 1,000 reproductive age women. For LARC (IUD and implants) and tubal ligation we calculated the cumulative uptake per 1,000 reproductive age women. All calculations were repeated using both the TBBC and UNHR population estimates.

### Ethics statement

For the extraction of data, ethical approval for retrospective analysis of pregnancy records was given by the Oxford Tropical Research Ethics Committee (OXTREC 28–09, amended 19 April 2012).

## Results

### Population estimates

**Fig 1:**
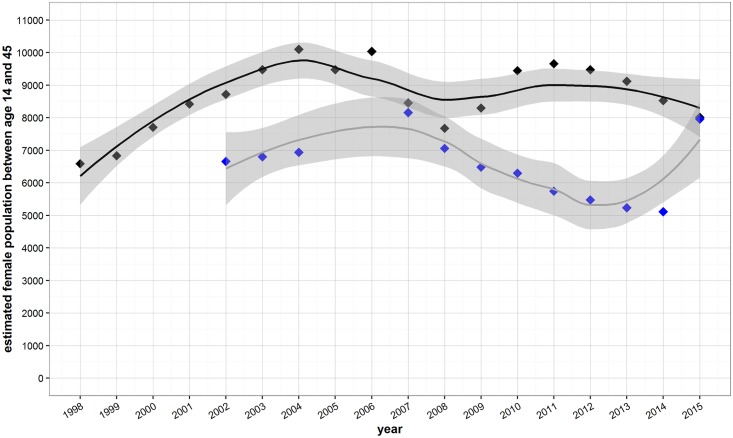
Estimated reproductive age female population for Maela camp by year and organization (filled diamonds) reporting population counts (TBBC or UNHCR). A loess curve (solid line) is fit to the data points and 95% confidence intervals for the curve are shown in dark gray.

Population estimates for Maela camp are vastly different by reporting agency, with UNHCR-based estimates being consistently smaller than TBBC estimates (Fig 1) for most years. The two agencies showed congruence in population estimates in 2007 and 2008, then moved in opposing directions with TBBC showing population estimates levelling off and UNHCR showing depopulation, and finally converging again in 2015.

### New contraceptive consultations over time

From 1996 through 2015 SMRU and PPAT collectively recorded 35,139 new family planning consultations (Table 1). In 2011 there was a notable drop of approximately 28% new contraceptive users compared to 2010 (Table 1). However, the trend of increasing consultations per 1,000 reproductive age women remains. The number of new contraceptive consultations per 1,000 reproductive age women in 2002 using TBBC population estimates was 156.7 (95% CI: 149.2–164.4) and by UNHCR estimates was 218.8 (95% CI: 208.8–229.1). By 2015 the number of new contraceptive consultations per 1,000 reproductive age women had increased to 200.3 (95% CI: 191.8–209.1) by TBBC population estimates and 226.9 (95% CI: 217.4–236.7) by UNHCR population estimates.

**Table 1:** New family planning consultations by year and contraceptive type by PPAT and SMRU.

Year	injection	pill	condom	implant	IUD	tubal ligation	vasectomy	total
1996	39	9	0	5	0	6	0	59
1997	265	54	11	95	8	16	8	457
1998	311	118	45	22	11	15	14	536
1999	452	98	33	7	62	8	53	713
2000	550	132	490	21	11	31	34	1269
2001	731	256	573	56	12	71	93	1792
2002	540	207	295	162	33	59	117	1413
2003	547	306	279	55	66	67	24	1344
2004	551	351	238	3	41	88	34	1306
2005	547	453	285	69	32	125	11	1522
2006	580	510	338	108	38	257	6	1837
2007	631	637	297	92	54	202	4	1917
2008	614	703	260	103	26	60	8	1774
2009	1116	1500	545	83	50	93	13	3400
2010	1225	1409	624	64	70	62	7	3461
2011	982	1023	301	30	67	89	1	2493
2012	1191	1336	486	72	109	84	4	3282
2013	902	1118	338	38	92	95	4	2587
2014	1001	814	297	43	99	61	0	2315
2015	663	513	123	6	267	90	0	1662
								**35139**

### Contraceptive uptake by type and year

**Fig 2:**
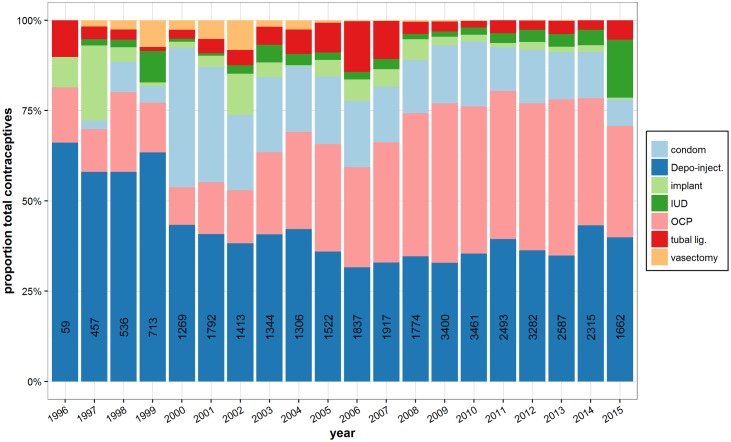
Composition of total recorded new contraceptive consultations by contraceptive type and year. The total number of new consultations per year is indicated in each bar. There was a transition in most reproductive health services from SMRU to PPAT in 2000. The increase proportion attributable to tubal ligation (“tubal lig.”) in 2005–2006 coincides with SMRU hiring a local surgeon who could offer the service post-partum.

**Fig 3:**
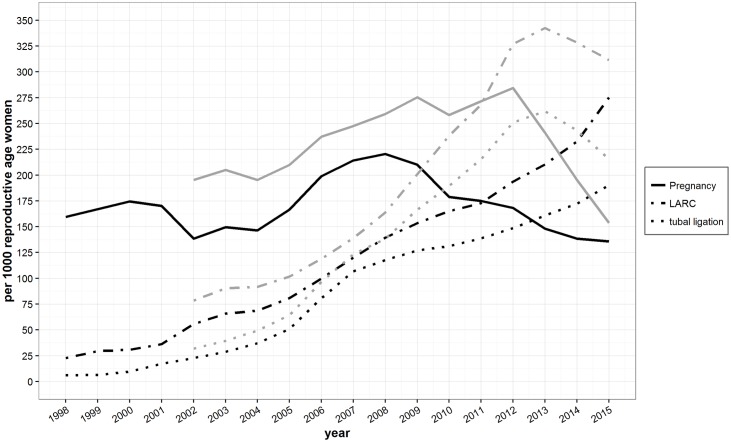
Cumulative uptake of long acting reversible contraceptives ((LARC) implants and IUD) and tubal ligations, and pregnancies per 1,000 reproductive age women using different population estimate origins (TBBC (black lines) or UNHCR (gray lines)) by year.

Trends in contraceptive uptake were compared over time (Figs 2 and 3). Depo-injections, pills, and condoms have been the three major contraceptive choices in the camp (Fig 2). Depo-injections composed over 50% of all new contraceptives consultations until 2000 and remained the most popular method among new contraceptive clients in the camp until around 2008 when the pill began to make up approximately an equal proportion of new contraceptive type. Condom uptake from the clinic decreased. ECPs do not feature because there were no requests from clients for this method.

### Cumulative uptake of long-acting and permanent methods

IUD uptake has slowly increased throughout the time period (Fig 3). In 2002 cumulative IUD uptake was around 15 or 25 per 1,000 reproductive age women whereas in 2012 it was either around 125 or 75 per 1,000 reproductive age women (TBBC- and UNHCR-based population estimates respectively). Over the last decade, implant uptake appears to have at least doubled (TBBC-based population estimates) and potentially quadrupled (UNHCR-based population estimates). Female sterilizations (tubal ligation) followed the same general pattern as LARC until 2004 when they began to increase and surpassed LARC.

The cumulative uptake estimates here are sensitive to the underlying population estimates; however a spike in the uptake of tubal ligations between 2004 and 2007 is apparent regardless of the denominator. Based on both TBBC and UNHCR population estimates the cumulative uptake of tubal ligation more than quadrupled between 2002 and 2007. If UNHCR population estimates are correct, the uptake of tubal ligations continued on this upward trajectory, potentially increasing over 10 fold between 2002 and 2014 (with approximately 27% of all reproductive age women having had the procedure), whereas by TBBC estimates it has increased slowly since 2007.

### Pregnancy and miscarriage rates

As with the cumulative incidence of LARC uptake and tubal ligation, pregnancy and miscarriage rates are sensitive to the underlying denominator (Fig 3). However, general trends are apparent regardless of the different population estimates. The pregnancy rate is currently decreasing. Based on UNHCR population estimates, pregnancy rates increased by almost 43% between 2002 and 2012, from 195 to 284 pregnancies per 1,000 reproductive age women. Using TBBC population estimates, pregnancies appear to have undergone an initial decrease (by almost 13%) between 1998 and 2002 (160 to 138 births per 1,000), an 36% increase from 2004 to 2006 (147 to 199 pregnancies per 1,000) and then another period of decrease from 2009 through 2015, reaching a slightly lower pregnancy rate than in any other recorded year (137 pregnancies per 1,000 reproductive age women).

**Fig 4:**
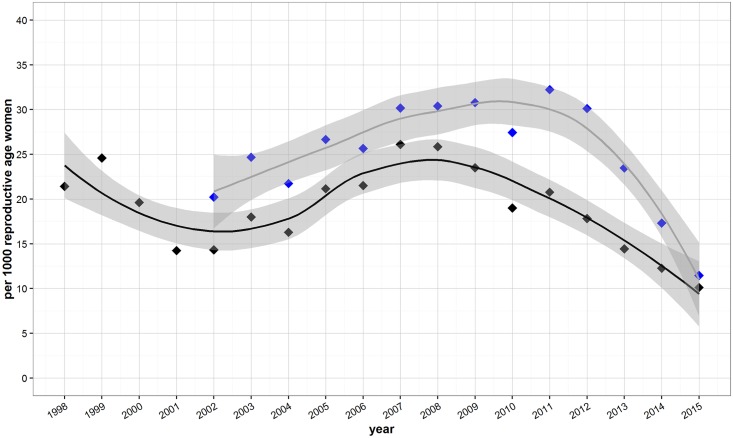
Miscarriages per 1,000 reproductive age women over time. Blue points are rates calculated using UNHCR population estimates and black points are from estimates using TBBC population estimates. A loess curve (solid line) is fit to the data points and 95% confidence intervals for the curve are shown in dark gray.

Miscarriage rates showed small fluctuations over time but remained relatively stable for most of the time period (Fig 4). Using TBBC population estimates the miscarriage rate in 1998 was 21 per 1,000 women. It fluctuated over the time period, decreasing to approximately 15 per 1,000 women in 2001 and increasing back to 25 per 1,000 women in 2007. Using UNHCR population estimates the rate began at around 20 per 1,000 reproductive age women in 2002, increased to 30 per 1,000 by 2007 and began a decrease in 2011. There is an apparent decrease in recent years, with the most recent estimates (from 2015) of miscarriage rates indicating 10 or 11.5 per 1,000 women using TBBC or UNHCR estimates respectively.

## Discussion

Our results indicate encouraging trends with regard to family planning in Maela refugee camp. LARC uptake appears to be increasing while pregnancies and miscarriages are decreasing (Figs 3 and 4). Pregnancy rates remain relatively high in the camp [31]. While pregnancy rates are high, the increased uptake of LARC and permanent methods coincide with decreases in maternal and neonatal mortality [32,33], though we are cautious in linking these trends because this analysis has not accounted for other mortality-related factors and the data do not allow us to draw causal relationships. Previous linkage between crude birth rates and mortality in refugee and internally displaced persons suggested reproductive health outcomes could be better in post-conflict phases than in the host country or in the country-of-origin [34].

The pattern of change reported here roughly follows the demographic transition model (with an initial decrease in mortality followed by a decrease in fertility) which has been observed in several regions of the world as populations undergo economic development [35,36]. Mortality data for refugees in relation to family planning services is frequently limited. Burundi refugees included in the 1988 census data in Tanzania had a 6 fold higher risk of child death compared to Tanzanian mothers who were at the highest risk of child bearing; the highest risk Tanzanian mothers having a child death was 6.4 fold higher than low risk Tanzanian mothers [37]. This emphasizes the need to provide family planning early on during refugee settings, when mortality is usually at its highest [38], when populations may be fluctuating most, and when the demographic and health provision situation may be most chaotic.

The stark increase in the uptake of female sterilization beginning in 2005 (Fig 3) coincides with employment of a surgeon by SMRU (2005–2007) who could provide tubal ligation immediately post-partum. Uptake was high because the doctor lived in the camp full time and could fluently speak the local camp languages (both S’gaw Karen and Burmese) allowing adequate explanation and strong doctor-patient relationships [39]. Local misconceptions and fears surrounding operations could be addressed directly rather than through an interpreter [40].

Miscarriages appear within the ranges reported from developed nations [41,42] and have remained relatively stable over time (Fig 4), with recent data suggesting declining rates. At the same time, ECPs uptake has been extremely low in the camp, despite education efforts geared toward both camp dwellers and family planning staff [43]. Karen culture is conservative with regard to intercourse and pregnancy [23,44] and low ECP uptake could be explained by socio-cultural factors; by misconceptions about ECPs; or perhaps by women obtaining ECPs outside the camp [43]. The recommendation by Hobstetter et al for “increased outreach and education campaigns to raise community awareness and demand for ECPs” still stands but requires an appropriate level of funding, cooperation between agencies and addressing of misconceptions [43].

In conflict settings, decisions must be made about how and where to allocate limited funds [45] and reproductive health is not always prioritized. Changes in contraceptive uptake in Maela camp (Figs 2 and 3) are directly related to donor funding. For example, low literacy amongst the female population [46] required resources to be channeled toward training staff who speak local languages (S’gaw Karen, Pwo Karen and Burmese) in order to provide verbal or pictorial information and counseling to women. Qualified personnel (such as the previously mentioned surgeon) that could provide permanent contraception in a socio-culturally appropriate manner also required funds [39]. Free provision of contraceptives in the refugee camp probably contributed positively to uptake but data on the impact of patient donations was poor. Fluctuations in funding resulted in shifts in health services and the contraceptives that could be provided. This is well described in relation to other camp services by Cottrell [27] with regard to camp management and the provision of food. Likewise, the lower rate of implant uptake in 2000–2001 was related to the end of the FPA funding and in 2015 a decision was made to primarily provide IUD (since it permits at least 4 times more women to be reached compared to implants as the cost was 600 versus 2,200 Thai baht in the same year) because of the small family planning budget. Similar patterns are documented from other regions. For example, contraceptive uptake by Afghan refugee women living in Pakistan was observed to be directly associated with subsidized healthcare [47]. The call for agencies and governments to maintain funding for free family planning and other reproductive health services for refugees, internally displaced and marginalized populations is far from a new concept [48]. Recent massive population displacements, stemming from conflict in the Middle East [3] have refocused the spotlight on this important topic.

Contraceptive uptake in Maela camp is dominated by Depo-injections and the COCP (Fig 2). In the past, implant had higher uptake than did IUD but IUD uptake has consistently increased in the camp despite the slower uptake in different areas along the Thailand-Myanmar border [49] and Malaysia-Myanmar border [50]. In 2015 it was not possible to discern if higher IUD uptake was due to a required client donation for the implant or to uncertainty about the future as plans for voluntary repatriation of the camps move forward [51].

There are several limitations to this work. Foremost among these limitations are conflicting census estimates for Maela refugee camp. We have attempted to address (and highlight) this problem by using both UNHCR and TBBC population estimates as denominators. In relation to family planning programs in conflict settings Curry *et al*. [16] suggested the avoidance of costly population surveys and suggested that providers should focus on obtaining ‘high quality routine monitoring data’, using this to improve programs. The five sites studied by Curry et al all worked through the Ministry of Health unlike the situation in Maela camp [16]. In Maela camp census attempts and family planning uptake are obfuscated by the chaotic environment, complicated political situations, population fluctuations (frequently from new conflicts or skirmishes), limited funding and human resources. In places where the reproductive health needs are the greatest, the extent of those needs may be most difficult to accurately document and report, potentially leading to a cycle of public health and humanitarian neglect or misdirection of services [52].

## Conclusion

The data presented here suggest that efforts to provide contraceptives in refugee settings are likely to yield benefits and that women and men, voluntarily seek such services when they are provided in an economically and socio-culturally appropriate manner. Unintended pregnancies represent unanticipated and largely avoidable social, economic, and health costs to women, couples, families and communities. Contraceptive services have significant and important benefits for maternal health and for communities [53] and humanitarian efforts should include contraceptives (not only ECPs). Funding agencies should give careful consideration with regard to the provision or withdrawal of family planning funding in humanitarian settings. Finally, data collection is a major problem in humanitarian settings and there is a great need for innovative approaches of collecting demographic and epidemiological indicators, given inherent complications as well as limited and fluctuating funding.

## Supporting information

S1 Table Estimated reproductive age women (15–44 years old) based off of TBBC and UNHCR reports; smoothed estimates from loess curves; and counts of births and miscarriages in Maela camp women. Estimations are described in detail in the Analysis section.
(XLSX)
